# Discrepancies in Mineral Oil Confirmation by Two-Dimensional Gas Chromatography–Mass Spectrometry: A Call for Harmonization

**DOI:** 10.3390/molecules30132830

**Published:** 2025-07-01

**Authors:** José Fernando Huertas-Pérez, Cristina Cruz-Hernández, Antonio Núñez-Galindo, Mathieu Dubois, Loïc Perring, Adrienne Tarres, Julie Nicolay, Céline Vocat, Thierry Delatour

**Affiliations:** Nestlé Research, Société des Produits Nestlé S.A., Vers-chez-les-Blanc, CH-1000 Lausanne, Switzerlandceline.vocat@rd.nestle.com (C.V.); thierry.delatour@rdls.nestle.com (T.D.)

**Keywords:** mineral oil, food contaminants, GCxGC-ToF, LC-GC-FID, laboratory comparison

## Abstract

Three different vegetable oils, namely coconut oil, palm olein and olive oil, were analyzed for mineral oil hydrocarbons (MOHs) in our laboratory and in five commercial laboratories well recognized for their expertise in this field. The analysis consisted of a preliminary quantitative estimation of MOH content by hyphenated liquid chromatography–gas chromatography with flame ionization detection (LC-GC-FID), followed by a confirmatory analysis of MOH components by two-dimensional gas chromatography with time-of-flight mass spectrometry (GCxGC-ToF). The results provided by the six laboratories were compared to check their consistency, which would have led to a hypothetical commercial agreement or dispute scenarios, for instance. The comparison was based merely on information provided by the laboratories in their analytical reports (i.e., the methodology was not challenged, and chromatograms were not reviewed). Additionally, some of the laboratories were willing to provide some more information or details of the analysis. Similar quantitative results were provided by all six laboratories, emphasizing the utility of the current available harmonized guidelines and official standards for this method. However, as regards confirmatory results, discrepancies were observed among some laboratories in terms of the detection of MOH markers at low levels and the interpretation of GCxGC-ToF information. Even taking into account the limitation of this study as regards the reduced number of laboratories included, it highlights the need for harmonizing the GCxGC-ToF confirmatory method for MOHs in order to increase the alignment of results between laboratories for this kind of analysis.

## 1. Introduction

Food safety is of utmost importance for the food industry, particularly in the current era of raw materials and products that are traded globally [1]. Among various chemical compounds that may enter the food chain, hydrocarbons of mineral oil origin account for known contamination in foodstuffs [2,3]. They enter food by migration from materials in contact with food, from absorption into raw materials stored under inappropriate conditions or through negligence in farming and manufacturing practices. Mineral oils are obtained by petroleum refining and therefore contain a large number of different hydrocarbons such as alkanes (paraffins), cycloalkanes (naphthenes), aromatic hydrocarbons or asphaltenes, whose patterns derive from the paleoenvironmental conditions of the relevant crude oil [4,5,6]. Because of the complex composition of mineral oils, their analysis is a challenging task that requires complex sample preparation and non-standard hyphenated analytical techniques.

Today, liquid chromatography hyphenated to gas chromatography coupled to flame ionization detection (LC-GC-FID) is referred to as the method of choice for the quantification of mineral oils in routine analysis [7]. However, due to the high complexity of mineral oil composition, LC-GC-FID does not provide component resolution beyond the separation of mineral oil saturated hydrocarbons (MOSHs) and mineral oil aromatic hydrocarbons (MOAHs) [8,9,10]. In the past, the diversity in the conditions of sample preparation, together with the various ways of treating data and reporting results, used to lead to significant differences in levels reported by different laboratories [11,12]. This kind of situation leads to possible disruption across food operators and the erosion of confidence in industrial food products by consumers. In the European Union, the Joint Research Centre (JRC) has released a guidance document recommending best practices in the analysis of mineral oil hydrocarbons (MOHs) in food in order to prevent discrepancies in the results [13], which has been recently updated [7]. Such guidelines, setting the frame for analysis and method performances, have helped reaching consensus as regards the procedure, analytical conditions and reporting for the quantification of mineral oils. However, they will not overcome the inherent limitations of LC-GC-FID for addressing the challenge of mineral oil confirmation in food matrices. Furthermore, LC-GC-FID is not a confirmatory method for the control of chemical substances and the residues thereof, as defined by the EU regulation since 2002 [14].

To circumvent the lack of specificity of the flame ionization detector, comprehensive two-dimensional gas chromatography (GCxGC) has been proposed and seems to be the most promising solution available today, and the European Food Safety Agency (EFSA) recommends its use coupled to a time-of-flight mass spectrometer (ToF) for MOH confirmation [15]; thus, several commercial laboratories have implemented this technique for the confirmation of mineral oils. GCxGC coupled with a ToF makes use of the orthogonal principle of different column selectivity to improve the efficiency of the separation, together with structural identification provided by mass spectrometry. GCxGC-ToF technology has already demonstrated promising perspectives in the analysis of complex petrochemical mixtures [16,17,18], providing opportunities for the characterization and identification of specific classes of compounds in the mixtures. In food control, GCxGC-ToF has shown to provide sufficient selectivity for resolving different aromatic ring systems [19], thereby helping food operators to perform root-cause analysis and ultimately take adequate actions for a supply chain free of any hazardous mineral oils.

Nonetheless, the interpretation of data generated by GCxGC-ToF, usually depicted on a two-dimensional map with numerous spots color-coded according to signal intensity, requires deep expertise to draw adequate conclusions on each sample. Signals present in the procedural blank sample and overlapping in chemical classes may lead to erroneous interpretation if insufficient attention is paid to these aspects.

The present study aimed to assess the consistency of results obtained by six different laboratories well recognized as experts in the analysis of MOH, which were requested to quantify and confirm mineral oil components in three different oil samples, namely coconut oil (CO), palm olein (PO) and olive oil (OO). The selected samples were analyzed by LC-GC-FID prior to GCxGC-ToF for the quantification of the MOSH and MOAH fractions. The results provided by the six different laboratories were compared based merely on the information provided by the laboratories in their analytical reports (i.e., the methodology was not challenged, and chromatograms were not reviewed). Additionally, some of the laboratories were willing to provide some more information or details of the analysis performed. The objective was not to run an additional interlaboratory trial by LC-GC-FID (several trials are available in the literature [20,21]) but to show that similar results obtained by LC-GC-FID do not ensure the alignment of the results when the same samples are qualitatively analyzed by GCxGC-ToF. Ultimately, and despite the limitation of low commercial laboratories available for GCxGC-ToF analysis of MOH, the objective of the study was initially to check the degree of alignment among available laboratories equipped and skilled to perform the confirmatory analysis of MOH by GCxGC-ToF, and ultimately to raise awareness of the risk of erroneous conclusions if safety assessments are based on GCxGC-ToF results generated with inadequate reliability, thus highlighting the need for harmonizing the GCxGC-ToF confirmatory method for MOH.

## 2. Results and Discussion

### 2.1. Laboratory Comparison of Quantitative Results Generated by LC-GC-FID

The use of LC-GC-FID was proposed a decade ago for the quantification of MOH, and it is considered today the gold standard technique for this application [8,9]. It is based on the LC separation of the MOSH and MOAH fractions prior to further GC separation and quantification of unknowns by FID using one internal standard per fraction. Due to the complexity of the mixture, the chromatograms generated do not contain isolated peaks but a so-called unresolved complex mixture or ‘hump’, which requires experienced analysts to be integrated properly. As regards sample preparation, the method involves, for some matrices (including edible oils), a clean-up step based on an epoxidation reaction, which also requires deep experience due to risks of the incomplete elimination of interferences or loss of MOHs [22]. In the European Union, recent efforts were deployed to reach a consensus optimized procedure intended to prevent extensive discrepancies between laboratories that control mineral oils in food products [7,23].

All laboratories reported quantitative data of MOSH and MOAH fractions, obtained by LC-GC-FID, and reported per carbon fraction, as well as total MOSH/MOAH, as specified in the first edition of the JRC guidelines [13]. Measurement uncertainties in the 30–40% range were also reported, depending on the lab, which are accepted for this analysis considering its complexity [7]. Results are shown in Table 1 and Table 2 for MOSH and MOAH, respectively. Differences were observed between LOQs claimed by labs, ranging from 0.4 to 2 mg/kg. In some cases, LOQs were increased for specific samples due to the presence of interference and therefore lower method reliability at low levels. This was the case of laboratory C for the MOAH fraction of CO (increased LOQ from 1.0 to 2.0 mg/kg for carbon fractions C25–C35 and C35–C50) and Lab E for the MOAH fraction of the OO sample (from 0.5 to 2.8 mg/kg for total MOAH). Each set of data was checked for outliers by means of the Grubbs’ test (significance level α = 0.05). No outliers were found, apart from the total MOAH value of Lab B in palm olein (*p* < 0.05). As can be seen in Table 1 and Table 2, generally comparable quantitative results were reported for the MOSH and MOAH fractions, although some misalignments were found. For instance, as regards the MOSH fraction, light mismatching was found between the results of laboratory B and those reported by laboratories D/E for CO (C25–35, C40–50 and total MOSH) and PO (C40–50 and total MOSH). In the case of MOSH in OO, misalignments were more evident for all carbon fractions and total MOSH between the lower and higher reported values. As regards the MOAH fraction, most of the misalignments were caused by the inability of some laboratories to carry out quantification at low levels for specific matrix/carbon range combinations (probably because of a significant amount of biogenic interferences at those low concentration levels). Nevertheless, these results showed a significant improvement over the previous situation, when different studies evidenced differences in quantitative MOH results that could exceed 40-fold for MOAH in cocoa butter or MOSH in biscuits. The range was even broader for MOSH in infant formula, with a 143-fold difference observed between the highest level measured (4.3 mg/kg) and the lowest one, reported to be <0.03 mg/kg [12]. Although the number of laboratories and matrices included in this study are limited, the obtained results are in line with what was expected beforehand after all the effort carried out during the last 5 years by all stakeholders, including the standardization of organisms, authorities, industries and testing laboratories to have a harmonized quantitative method for MOH analysis, including all the stages, such as sampling, analytical methodology, method performance, data treatment and reporting [7,23,24]. This certainly allowed reducing the high variability of the results regularly generated by laboratories only a few years ago [12].

### 2.2. Laboratory Comparison for the Confirmation of Mineral Oil Components by GCxGC-ToF

In line with EFSA recommendations as regards digging deeply into the composition and better characterization of MOH, by monitoring on the one hand compounds containing different number of aromatic rings in the MOAH fraction, and on the other hand the interferences in the MOSH fraction, such as polyolefin oligomeric saturated hydrocarbons (POSHs) or polyalphaolefins (PAOs) [15,25], GCxGC-ToF is the technique of choice for the confirmation of mineral oil markers in samples previously quantified by LC-GC-FID, or when more information about the different types of structural sub-groups is needed [19,26]. However, less experienced laboratories struggle to implement the technique because of the lack of guidelines on minimum instrument performance and interpretation of results, as well as the lack of means of comparing results obtained among themselves. Luckily, the situation is slowly changing with the emergence of some commercially available ring trials [27,28].

Three samples of different vegetable oils contaminated with MOHs and quantified by LC-GC-FID were dispatched to each laboratory for the confirmation of MOH markers by GCxGC-ToF analysis. Each participant provided a list of detected markers of MOHs, which were categorized according to chemical classes. The results are summarized in Table 3. Lab A did not perform the identification of compounds observed in the MOSH fraction, while all others reported species in both fractions.

**Figure 1:**
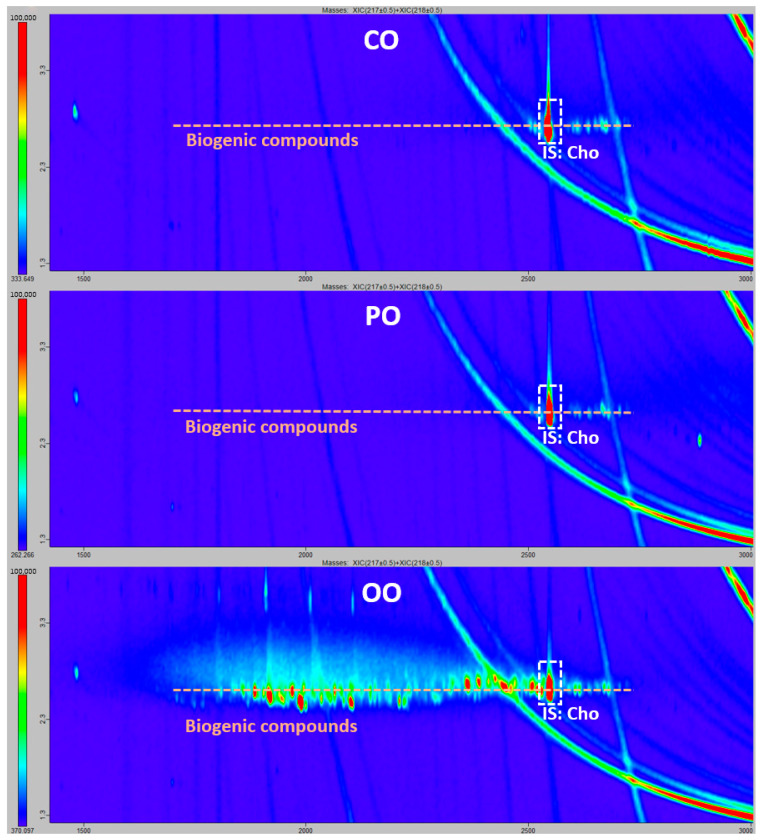
Extracted ion GCxGC-ToF chromatogram: *m*/*z* 217, 218 typical ions used in laboratory F to monitor biogenic compounds; IS: Internal standard; Cho: 5-α-cholestane.

Apart from Lab A, which did not report on the MOSH fraction, all labs confirmed the presence of mineral oils by reporting detected markers, such as hopanes, in the three samples. Interestingly enough, despite detecting hopanes (unequivocal markers of MOH), Lab E did not report any other MOH chemical class on the MOSH fraction. All the other labs reported comparable results for n-alkanes, iso-alkanes and cycloalkanes that were detected in the MOSH fraction of the three oil samples. As regards potential interferences, POSHs were evidenced by Lab E in CO and OO and by Labs B, E and F in PO. In our opinion, only PO samples contained traces of POSH, and the low amount is the reason for the disagreement between labs for these samples, while the results reported by Lab E for CO and OO should be considered as false positives. All labs, except Lab E reported biogenic compounds in the MOSH fraction on CO, and only two in PO and OO (Labs C and F). Figure 1 shows the extracted ion chromatograms (*m*/*z* 217, 218) for the three samples obtained in laboratory F for the MOSH fraction. As can be seen, the presence of biogenic compounds in OO is quite evident, while in the other two samples, those interferences were in a much lower concentration, which increased the discrepancy rate between laboratories.

**Figure 2:**
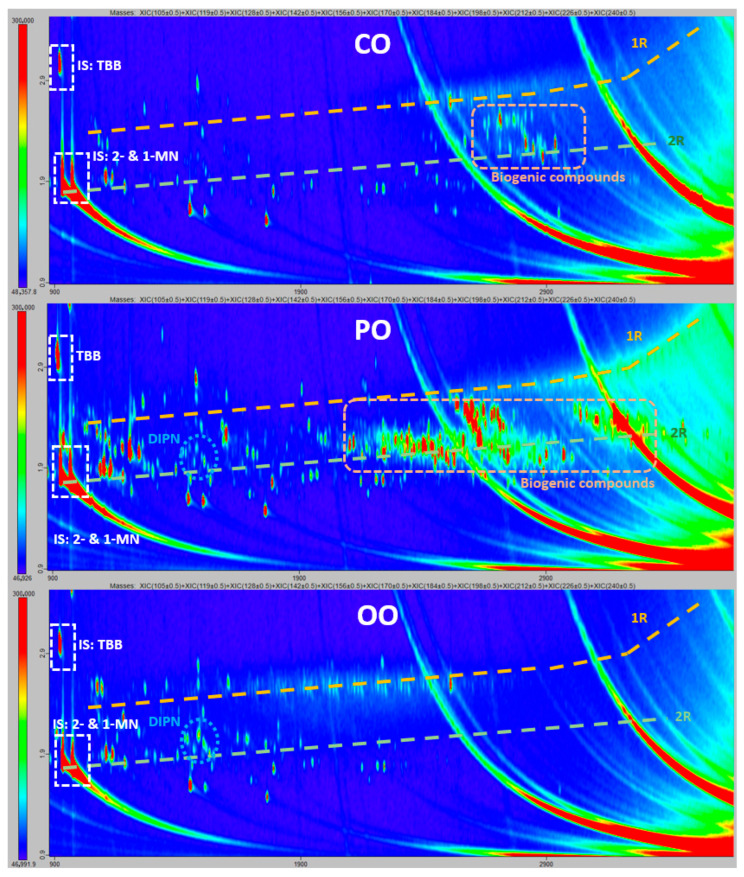
Extracted ion GCxGC-ToF chromatogram: *m*/*z* 105, 119, 128, 142, 156, 170, 184, 198, 226, 240, typical ions used in laboratory F to monitor alkylated mono- and di-aromatic ring systems; IS: Internal standard; TBB: 1,3,5-Tri-*tert*-butylbenzene; 1- and 2-MN: 1- and 2-Methylnaphthalene; 1R: mono-aromatics; 2R: di-aromatics; DIPN: di-isopropyl-naphthalene.

**Figure 3:**
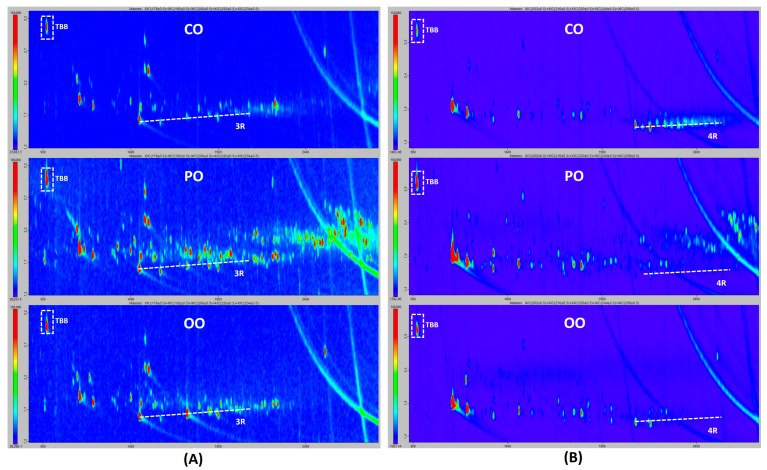
Extracted ion GCxGC-ToF chromatogram: (**A**) *m*/*z* 178, 192, 206, 220, 234, typical ions used in laboratory F to monitor alkylated tri-aromatic ring system; (**B**) *m*/*z* 202, 216, 230, 244 and 258, typical ions used in laboratory F to monitor the alkylated tetra-aromatic ring system; TBB: 1,3,5-Tri-*tert*-butylbenzene; 3R: tri-aromatics.

In the MOAH fraction, some discrepant results were reported for alkylated monoaromatic hydrocarbons, which were detected and identified by most of the participants in the three oil samples, except Lab E across the set of three oils tested, and Lab D in CO. Conversely, Lab E did report alkyl-diaromatics on the three samples. The composition of mineral oils containing two-ring MOAH with the absence of one-ring MOAH is highly unusual. Therefore, these results (the absence of one-ring MOAH) were considered as a false negative. Discrepant results were also obtained for alkylated diaromatics and the 3–7-ring system MOAHs. While GCxGC allows the separation of different MOH chemical classes and drawing chromatographic regions for each of them, some of these regions may overlap across their limits, meaning that compounds of different sub-groups may coelute on those region limits, depending on the chemical structure. Therefore, differences in chromatographic interpretation and/or lack of performance at low contamination levels, could hypothetically be the reason for some labs missing ring system MOAHs with two or three or more rings. Although GCxGC-ToF provides only pure qualitative results, as can be seen in Figure 2, concentration levels of two-ring MOAHs seem to be lower than those of biogenic compounds in CO and PO samples. These two classes of compounds coelute in the less volatile chromatographic region, which may cause chromatogram misinterpretation. This was evidenced by the fact that Labs A and E did not report any biogenic compounds in CO and PO, while being the only labs reporting alkyl-diaromatics in CO. The presence of biogenic compounds was highlighted by the same four laboratories, in CO and PO, essentially. In CO, they were assigned to triterpenes (Lab B), terpenes and steroids (Lab C) or flagged without detailed identification (Labs D and F). In PO, Lab B identified clusters of sesquiterpenes, triterpenes and carotenes, while Lab C assigned the signals observed to terpenes and steroids. In the case of the OO sample, no biogenic compounds were reported by any laboratory, and alkylated diaromatics were reported by four laboratories (all except Labs A and B). Again, concentration levels for the last ones were very low, and the signals corresponding to this MOH chemical class were discrete peaks (i.e., the di-isopropylnaphthalenes cluster and other alkylated naphthalenes) rather than a cloud of isomers, which is the usual kind of signal obtained for MOH [19]. Therefore, the inadequate sensitivity of the method, or the subjective judgement of the analysts in reporting trace levels of contamination were hypothetically the reasons for those different reporting results. The reasons for discrepancies as regards three- and four-ring system MOAHs were the same. From chromatograms from Lab F, shown in Figure 3, it can be observed that few discrete peak signals were obtained for those chemical classes in all samples, except for tetra-aromatics, for which a cloud was obtained. Polyaromatic hydrocarbons with five to seven rings were consistently not reported except for a single participant (Lab D) in all three samples. We observed extremely low discrete signals for a few five-ring PAHs only for CO samples.

Benzothiophenes and allied compounds were found in the three samples by a single participant (Lab E). Not any other participant in the trial pointed out the presence of this class of sulphur aromatic compounds. The epoxidation reaction applied as clean-up of the MOAH fraction during the sample preparation is known to eliminate thiophenes [22], and it should be carried out carefully under extremely controlled conditions to avoid losses of MOH markers. Therefore, this result may be correct despite being perceived as a false positive at first glance.

In summary, differences in GCxGC-ToF results can be attributed to lower performance at low concentration levels (i.e., laboratories missing steranes and POSH in the MOSH fraction), which may be caused by a less efficient sample preparation procedure or lower instrumental performance. In other cases, for instance, those differences were clearly due to different ways of interpreting chromatographic and mass spectra information, and different analysts’ criteria as regards reporting discrete peak signals (and not clusters of isomers) as MOH contamination.

## 3. Materials and Methods

### 3.1. Design of the Interlaboratory Trial

Six European commercial laboratories were selected for the interlaboratory trial; three of them were from Germany (Labs A, B and C), one laboratory was from Italy (Lab D) and two were from Switzerland (Labs E and F). Laboratory F is Nestlé Research Lausanne, Switzerland. Three samples of vegetable oils that contained different levels of mineral oil hydrocarbons (MOHs) were considered for the trial. They consisted of one CO, one PO and one OO, which were analysed by LC-GC-FID for the determination of MOSH and MOAH content prior to shipment to the participants for GCxGC-ToF analysis. Each participant was requested to determine MOSH and MOAH content (LC-GC-FID) prior to the identification of individual compounds or classes by GCxGC-ToF.

Samples distributed for the interlaboratory trial

Oil samples (CO, PO and OO) were sourced from our own facilities, selected for this study based on their MOH content and aliquoted in-house (20 g) into 50 mL polyethylene terephthalate glycol-modified (PET-G) bottles for distribution to the different laboratories.

### 3.2. Quantification of MOSH and MOAH by LC-GC-FID

As regards sample preparation, Labs A-D and F clearly mentioned in their reports that sample preparation and quantification by LC-GC-FID were based on the revised EN 16995:2017 [23] (now published under ISO 20122:2024 standard [29]) with modifications to decrease the LOQ and improve accuracy. To summarize, the method involves an initial hexane/ethanol distribution of the sample for the removal of interferences, followed by a saponification step with potassium hydroxide to remove lipids from the extract. A portion of the extract was epoxidized (of polyenes naturally present in oil samples) to avoid interference in the quantification of the MOAH fraction. The epoxidation can be carried out either off- or online by using the LC-GC-FID platform [23,30].

All labs applied LC-GC-FID for the quantification of the MOSH and MOAH fractions, according to the JRC guidelines [13]. Labs A, C, D and F used a LC-GC-FID platform supplied by Axel Semrau (Sprockhövel, Germany); Lab B a platform supplied by Gerstel GmbH & Co. KG (Mülheim an der Ruhr, Germany); and Lab E a platform supplied by Brechbüehler AG (Schlieren, Switzerland).

Labs B, D and F also mentioned in their reports that they followed the JRC guideline on sampling, analysis and reporting for the monitoring of MOH [13]. All other labs’ reports were in line with this guideline but did not explicitly mention it.

### 3.3. Confirmation of Mineral Oil Components by GCxGC-ToF

All labs applied GCxGC-ToF for the confirmation of MOH markers. Before injection into the GCxGC-ToF platform, all labs fractionated the sample extract into MOSH and MOAH using normal-phase LC and independently injecting those fractions offline, with the exception of Lab E, which used a hyphenated LC-GCxGC-ToF platform for direct online MOSH/MOAH fractionation and analysis by GCxGC-ToF. Platforms used by Labs A, C, D, E and F were from LECO (St. Joseph, MI, USA) consisted of an Agilent GC (Agilent Technologies, Santa Clara, CA, USA) coupled to a TOF Pegasus 4D BT (LECO, St. Joseph, MI, USA). The platform used in Lab B was supplied by Brechbüehler AG (Schlieren, Switzerland) and consisted of a GC chromatograph from Thermo Scientific (Waltham, MA, USA), a mass spectrometer from JEOL (Akishima, Tokyo, Japan) and a cryo-modulator from Zoex (Houston, TX, USA).

All labs used a reverse-phase column configuration on their GCxGC (mid-polar column on the first dimension and non-polar column in the second dimension), as it is usually recommended for this application [19,31,32,33]. Therefore, in all cases, equivalent elution patterns of MOH components are expected.

As thoroughly explained elsewhere [19,33], the interpretation of obtained chromatograms is qualitative, and it relies on the comparability of plots from samples and reference materials. On one hand, different MOH components can be identified by their elution pattern (elution region within the GCxGC and shape of signal clusters). Extracted ion chromatograms usually help the analyst to highlight certain markers that would otherwise be hidden or appear overlaid on the total ion chromatogram; therefore, the use of a list of typical *m*/*z* ratios of MOH markers for such monitoring is advisable. On the other hand, contaminants can be characterized and confirmed using spectral information by comparison with spectra stored in a reference base data, which can be commercially obtained (i.e., NIST, Wiley, etc.).

## 4. Conclusions

The results obtained for the quantification and confirmation of MOH by LC-GC-FID and GCxGC-ToF in edible oils, namely CO, PO and OO, were compared to check the consistency among six different laboratories. MOHs are very complex mixtures, and analytical methodologies for their monitoring are very complex. Therefore, measurement uncertainties in the 30–40% range are usually accepted for their quantitation. Consistent quantitative results were generally obtained among the six laboratories, with some light misalignment; however, considering the complexity of the analytical methodology and the uncertainties usually accepted, the quantitative values provided could be considered consistent. Additionally, they also showed a significant laboratory alignment improvement when compared with previous studies, as per the results of currently available guidelines and standards.

EFSA recommends digging deeply into the composition and better characterization of MOHs by monitoring and collecting data about different number of aromatic rings in the MOAH fraction, specifically 3 to 7 rings, which are associated with genotoxicity and carcinogenicity, and also by discriminating interferences in the MOSH fraction such as polyolefin oligomeric saturated hydrocarbons (POSHs) or polyalphaolefins (PAOs). GCxGC-ToF plays a key role, as it is the technique of choice to carry out a qualitative and confirmatory monitoring of MOH in line with EFSA recommendations. Therefore, the alignment of results provided by laboratories for this analysis is also of utmost importance. In the frame of this study, misalignments were observed among the GCxGC-ToF confirmatory results reported by the six different laboratories due to either sub-performance, different reporting criteria or incorrect data interpretation. Despite the limitation of a low number of laboratory results included in this study, these results highlight the need to harmonize the confirmatory analytical method for MOH in food based on GCxGC-ToF. There have been certain efforts in this direction, like the recent offer of some proficiency tests (PTs; three editions; one PT/year) by one supplier, although with a very limited number of participants (5–8 laboratories). In order to have comparable results across different laboratories, it is key to start working groups to develop guidelines and official standards specifying the minimum performance criteria, describing how to perform the analysis to establish identification criteria for key MOH markers, defining reporting thresholds for trace signals and setting guidance on when to report either clusters or discrete signals. The availability of more PTs and the broader participation of laboratories in them is also recommended.

## Figures and Tables

**Table 1:** Quantitative results for MOSH * fraction (mg/kg) obtained by LC-GC-FID.

Vegetable Oil	Laboratory	MOSH [mg/kg]													
		C10–C16	U	C16–C20	U	C20–C25	U	C25–C35	U	C35–C40	U	C40–C50	U	Total	U
Coconut oil	A	<LOQ (1.0)	-	<LOQ (1.0)	-	<LOQ (1.0)	-	11.0	±4.4	5.0	±2.0	4.7	±1.9	20.7	±8.3
	B	<LOQ (0.4)	-	<LOQ (0.4)	-	0.8	±0.2	18.0	±5.2	7.1	±2.1	8.1	±2.3	34.0	±9.9
	C	<LOQ (1.0)	-	<LOQ (1.0)	-	<LOQ (1.0)	-	14.0	±4.2	5.0	±1.5	5.1	±1.5	24.1	±7.2
	D	<LOQ (2.0)	-	<LOQ (2.0)	-	<LOQ (2.0)	-	9.2	±2.1	4.0	±1.0	3.2	±0.9	17.0	±4.0
	E	<LOQ (0.5)	-	<LOQ (0.5)	-	0.7	±0.2	8.8	±2.6	4.0	±1.2	3.9	±1.2	17.4	±5.2
	F	<LOQ (0.5)	-	<LOQ (0.5)	-	0.7	±0.2	11.8	±4.1	4.5	±1.6	3.9	±1.4	20.9	±7.3
	Median	-		-		0.7		11.4		4.8		4.3		20.8	
	SD					0.1		3.4		1.2		1.7		6.3	
Palm olein	A	<LOQ (1.0)	-	<LOQ (1.0)	-	<LOQ (1.0)	-	11.0	±4.4	9.5	±3.8	13.0	±5.2	33.5	±13.4
	B	<LOQ (0.4)	-	<LOQ (0.4)	-	0.5	±0.1	13.4	±3.9	12.1	±3.5	18.5	±5.4	44.5	±12.9
	C	<LOQ (1.0)	-	<LOQ (1.0)	-	<LOQ (1.0)	-	13.0	±3.9	9.9	±3.0	14.0	±4.2	36.9	±11.1
	D	<LOQ (2.0)	-	<LOQ (2.0)	-	<LOQ (2.0)	-	8.6	±2.0	7.6	±1.8	9.7	±2.2	26.0	±5.0
	E	<LOQ (0.5)	-	<LOQ (0.5)	-	<LOQ (0.5)	-	8.9	±2.7	6.8	±2.0	8.8	±2.6	24.5	±7.4
	F	<LOQ (0.5)	-	<LOQ (0.5)	-	<LOQ (0.5)	-	11.2	±3.9	8.5	±3.0	10.8	±3.8	30.7	±10.7
	Median	-		-		-		11.1		9.0		11.9		32.1	
	SD							2.0		1.9		3.5		7.4	
Olive oil	A	<LOQ (1.0)	-	<LOQ (1.0)	-	2.9	±1.2	9.4	±3.8	2.3	±0.9	1.5	±0.6	16.1	±6.4
	B	<LOQ (0.4)	-	0.8	±0.2	3.5	±1.0	13.3	±3.9	4.4	±1.3	3.4	±1.0	25.4	±7.4
	C	<LOQ (1.0)	-	<LOQ (1.0)	-	3.6	±1.1	12.0	±3.6	2.8	±0.8	1.9	±0.6	20.3	±6.1
	D	<LOQ (2.0)	-	<LOQ (2.0)	-	<LOQ (2.0)	-	6.7	±1.6	2.2	±0.6	<LOQ (2.0)	-	12.0	±3.0
	E	<LOQ (0.5)	-	<LOQ (0.5)	-	0.8	±0.2	5.1	±1.5	1.1	±0.3	0.7	±0.2	7.6	±2.3
	F	<LOQ (0.5)	-	1.2	±0.4	3.4	±1.2	15.5	±5.4	2.8	±1.0	1.4	±0.5	24.3	±8.5
	Median	-	-	-	-	3.4		10.7		2.6		1.5		18.2	
	SD					1.2		4.0		1.1		1.0		7.0	

LOQ: Limit of quantification. * MOSH fraction contains MOSH and analogues (such as polyolefin oligomeric saturated hydrocarbons; POSH). U: Expanded uncertainty (coverage factor, k = 2). SD: Standard deviation; average and SD were estimated when at least three values above the LOQ were available, and they do not include values below the LOQ.

**Table 2:** Quantitative results for MOAH fraction (mg/kg) obtained by LC-GC-FID.

Vegetable Oil	Laboratory	MOAH [mg/kg]									
		C10–C16	U	C16–C25	U	C25–C35	U	C35–C50	U	Total	U
Coconut oil	A	<LOQ (1.0)	-	<LOQ (1.0)	-	1.7	±0.7	2.1	±0.8	3.8	±1.5
	B	<LOQ (0.4)	-	<LOQ (0.4)	-	2.4	±0.7	3.2	±0.9	5.6	±1.6
	C	<LOQ (1.0)	-	<LOQ (1.0)	-	<LOQ (2.0)	-	<LOQ (2.0)	-	<LOQ (4.0)	-
	D	<LOQ (2.0)	-	<LOQ (2.0)	-	<LOQ (2.0)	-	<LOQ (2.0)	-	2.4	±0.7
	E	<LOQ (0.5)	-	<LOQ (0.5)	-	2.2	±0.7	1.8	±0.5	4.0	±1.2
	F	<LOQ (0.5)	-	<LOQ (0.5)	-	2.8	±1.0	<LOQ (0.5)	-	3.5	±1.2
	Median	-		-		1.95		2.1		3.8	
	SD					0.7		0.7		1.2	
Palm olein	A	<LOQ (1.0)	-	<LOQ (1.0)	-	1.3	±0.5	5.2	±2.1	6.5	±2.6
	B	<LOQ (0.4)	-	1.0	±0.3	4.0	±1.2	6.7	±1.9	11.7	±3.4
	C	<LOQ (1.0)	-	<LOQ (1.0)	-	2.3	±0.7	6.0	±1.8	8.3	±2.5
	D	<LOQ (2.0)	-	<LOQ (2.0)	-	2.0	±0.6	4.3	±1.1	6.5	±1.6
	E	<LOQ (0.5)	-	<LOQ (0.5)	-	2.2	±0.7	3.9	±1.2	6.1	±1.8
	F	<LOQ (0.5)	-	<LOQ (0.5)	-	1.7	±0.6	5.5	±1.9	7.2	±2.5
	Median	-		-		2.1		5.4		6.9	
	SD					0.9		1.0		2.1	
Olive oil	A	<LOQ (1.0)	-	<LOQ (1.0)	-	1.4	±0.6	<LOQ (1.0)	-	1.4	±0.6
	B	<LOQ (0.4)	-	0.7	±0.2	1.5	±0.4	1.0	±0.3	3.2	±0.9
	C	<LOQ (1.0)	-	<LOQ (1.0)	-	1.2	±0.4	<LOQ (1.0)	-	1.2	±0.4
	D	<LOQ (2.0)	-	<LOQ (2.0)	-	<LOQ (2.0)	-	<LOQ (2.0)	-	<LOQ (2.0)	-
	E	<LOQ (0.5)	-	<LOQ (0.5)	-	1.3	±0.4	<LOQ (0.5)	-	<LOQ (2.8)	-
	F	<LOQ (0.5)	-	<LOQ (0.5)	-	0.8	±0.3	<LOQ (0.5)	-	1.2	±0.4
	Median	-		-		1.3		-		1.3	
	SD					0.3				1.0	

LOQ: Limit of quantification. SD: Standard deviation; average and SD were estimated when at least three values above the LOQ were available, and they do not include values below the LOQ. U: Expanded uncertainty (coverage factor, k = 2). Data in red: outlier (Grubbs test, α = 0.05).

**Table 3:** Categorized compounds reported by the six participants of the trial in coconut oil (CO), palm olein (PO) and olive oil (OO) samples based upon GCxGC-ToF analysis.

Fraction	Sub-Group	Coconut Oil						Palm Olein						Olive Oil					
		Laboratory						Laboratory						Laboratory					
		A	B	C	D	E	F	A	B	C	D	E	F	A	B	C	D	E	F
MOSH	n-Alkanes		☑	☑	☑		☑		☑	☑	☑		☑		☑	☑	☑	☑	☑
	iso-Alkanes		☑	☑	☑		☑		☑	☑	☑		☑		☑	☑	☑		☑
	Cycloalkanes		☑	☑			☑		☑	☑	☑		☑		☑	☑	☑		☑
	Hopanes		☑	☑	☑	☑	☑		☑	☑	☑	☑	☑		☑	☑	☑	☑	☑
	Biogenic compounds		☑	☑	☑		☑			☑			☑			☑			☑
	POSH					☑			☑			☑	☑					☑	
MOAH	Alkyl-monoaromatics	☑	☑	☑			☑	☑	☑	☑	☑		☑	☑	☑	☑	☑		☑
	Alkyl-diaromatics	☑				☑		☑	☑		☑	☑	☑			☑	☑	☑	☑
	3–4 PACs	☑			☑		☑				☑		☑			☑	☑		☑
	5–7 PACs				☑		☑				☑						☑		
	Thiophenes					☑						☑						☑	
	Biogenic compounds		☑	☑	☑		☑		☑	☑	☑		☑						

☑: detected and reported. Blank: not reported.

## Data Availability

Data obtained in our laboratory are available upon request. Restrictions apply to the availability of chromatograms obtained from commercial laboratories and are not available due to their commercial policies.

## Abbreviations

The following abbreviations are used in this manuscript:

1- and 2-MN	1- and 2-Methylnaphthalene
1R	mono-aromatics
2R	di-aromatics
3R	tri-aromatics
C10–C16	Carbon range equivalent to an aliphatic chain with carbon numbers from 10 to 16
C16–C20	Carbon range equivalent to an aliphatic chain with carbon numbers from 16 to 20
C16–C25	Carbon range equivalent to an aliphatic chain with carbon numbers from 16 to 25
C20–C25	Carbon range equivalent to an aliphatic chain with carbon numbers from 20 to 25
C25–C35	Carbon range equivalent to an aliphatic chain with carbon numbers from 25 to 35
C35–C50	Carbon range equivalent to an aliphatic chain with carbon numbers from 35 to 50
C40–C50	Carbon range equivalent to an aliphatic chain with carbon numbers from 40 to 50
CO	Coconut oil
DIPN	di-isopropyl-naphthalene
FID	Flame ionization detector
GCxGC	Comprehensive two-dimensional gas chromatography
IS	Internal standard
LC-GC	Liquid chromatography hyphenated to gas chromatography
LOQ	Limit of quantification
MOAH	Mineral oil aromatic hydrocarbons
MOH	Mineral oil hydrocarbons
MOSH	Mineral oil saturated hydrocarbons
OO	Olive oil
PAO	Polyalphaolefins
PO	Palm olein
POSH	Polyolefin saturated hydrocarbons
ToF	Time-of-flight mass spectrometry analyzer
